# Pheromone-Trap Monitoring System for Pea Leaf Weevil, *Sitona lineatus*: Effects of Trap Type, Lure Type and Trap Placement within Fields

**DOI:** 10.3390/insects9030075

**Published:** 2018-06-27

**Authors:** Gadi V. P. Reddy, Govinda Shrestha, Debra A. Miller, A. Cameron Oehlschlager

**Affiliations:** 1 Western Triangle Agricultural Research Center, Montana State University-Bozeman, 9546 Old Shelby Rd, P.O. Box 656, Conrad, MT 59425, USA; govinda.shrestha@montana.edu (G.S.); debra.miller13@montana.edu (D.A.M.); 2 ChemTica USA, 2912 Enterprise Blvd. Durant, OK 74701, USA; cam@chemtica.com

**Keywords:** pheromone, insect, lure, trap, crop, pitfall trap

## Abstract

The pea leaf weevil, *Sitona lineatus*, is an important pest of field peas and faba beans worldwide. Present sampling techniques that rely on detection of adult feeding damage are labor intensive, time consuming and require repeated sampling. Semiochemical-based pest monitoring systems could improve pea leaf weevil management. This study, which was conducted in the Golden Triangle region of Montana, tested several factors that potentially might affect capture rates of pheromone-baited traps, including trap and lure type and trap placement. Pheromone-baited pitfall and ramp traps caught significantly more adults than ground or delta traps, in all study areas. Pitfall traps baited with gray rubber septa captured significantly more adults than traps baited with membrane formulations or controls in both pea and lentil fields. In addition, pheromone-baited pitfall traps positioned in the southern part of pea fields captured relatively higher numbers of adults than those placed in northern parts of fields, although this difference was not significant. These findings can be used to improve adult weevil monitoring and should be taken into consideration when developing an integrated pest management program.

## 1. Introduction

Field pea, *Pisum sativum* (L.) (Fabales: Fabaceae), is a significant pulse crop worldwide, and in the United States, 0.5 million hectares were planted in 2017 [1]. Presently, Montana is one of the leading pulse producers in the United States, ranking #1 in field pea, producing 48% of the USA. crop [2]. Field peas are grown as an annual crop for human consumption (whose benefits include high fiber, low fat, high protein, and low glycemic index) and their cultivation improves soil health and provides crop biodiversity [3].

The pea leaf weevil, *Sitona lineatus* (L.) (Coleoptera: Curculionidae), is a major pest of field peas and faba beans, *Vicia faba* (L.) (Fabales: Fabaceae), worldwide. This weevil is believed to be of European origin, but has spread to most field pea regions of the world, including Asia, Africa and North America, over the last 50 years [4,5,6,7]. In North America, this pest was first reported in 1936 by Downes [8]. In Montana, *S. lineatus* has been a serious economic pest of field pea in most of the state’s pulse growing region since 2010 [9].

*Sitona lineatus* is typically univoltine [10] and in the fall, adults migrate to field shelterbelts, alfalfa fields, and roadside areas where they feed on secondary leguminous hosts before overwintering in soil. In spring, when temperatures reach 12.5 °C, overwintered adults emerge and move into new plantings of field peas [11,12]. Oviposition, larval development, and pupation all occur in the soil. New adults emerge in late summer and migrate to secondary hosts to overwinter [12]. Adult feeding on pea seedlings leaves characteristic “U”-shaped notches along the leaf margins. Both larvae and adult feeding reduce photosynthesis, pod production, and formation of root nodules [12]. Larvae feed on the rootlets and on *Rhizobium* root nodules, causing weak root growth and decreasing nitrogen fixation [12]. In addition, larval feeding reduces seed protein content, particularly in nutrient-poor soils, as well as the amount of nitrogen returned to the soil [13]. It is believed that adults have less effect on yield than larvae, but this remains to be quantified [14]. El-Dessouki [15] reported that infestation of pea plants with 100 *S. lineatus* eggs per plant reduced yield by 27%.

In North America, pea leaf weevil management is currently based on the use of imidacloprid- or thiamethoxam-treated seeds, combined with foliar applications (often in combination with insecticide or fungicide products) if adult damage levels reach 30% (3 out of 10 plants along a seed row) during the 2nd to 6th node stages of field pea growth [9]. However, foliar insecticide applications alone have not been found to effectively protect yield [7,14]. In addition, current adult damage assessments are often labor intensive, time consuming, require repeated sampling and are not highly reliable as adult feeding is temperature dependent [16]. For this reason, the development of other pest monitoring methods, such as semiochemical-based traps, could improve pea leaf weevil management.

There is substantial worldwide interest in the development of a pheromone-trap monitoring system [17] and management program for *S. lineatus* [18,19,20,21,22,23,24]. Blight et al. [22] were the first to run bioassays on an adult male-produced aggregation pheromone of this weevil that was found to attract both males and females. Blight et al. [23] later isolated, identified, synthesized and assessed the attractiveness of the active component of this pheromone, 4-methyl-3,5-heptanedione. Field studies conducted worldwide have shown that this pheromone attracts both males and females in both spring and fall [21]. In addition, Landon et al. [24] showed that adults are also attracted to host plant volatiles in both spring and fall. The use of pheromone-baited traps offers a convenient and potentially potent tool for monitoring *S. lineatus* adult populations [18,19,20,21,22,23,24]. Work on pheromone traps for *S. lineatus* has been conducted since the pheromone compounds were first identified and being used for monitoring [18,19]. This trap allows early detection of the pest and allows differences in within-field pest infestations to be measured. The use of pheromone traps for this pest can improve decision making in pea leaf weevil management [18,19].

Insect capture in pheromone-baited traps can be influenced by several factors, including trap design (e.g., type, color, height and placement), lure type, pheromone dose, and environmental conditions during the trapping period [25,26,27]. Except for studies by Nielsen and Jensen [20] and by St. Onge et al. [21], that demonstrated that pheromone-baited cone and pitfall traps are effective in capturing adults, little information is available on how to optimize monitoring *S. lineatus* adults with pheromone traps. Here, we report field studies to assess the effects of trap and lure type and trap placement on adult pea leaf weevil catch to further enhance the trap efficacy for this pest.

## 2. Materials and Methods

### 2.1. Field Sites

**Figure 1:**
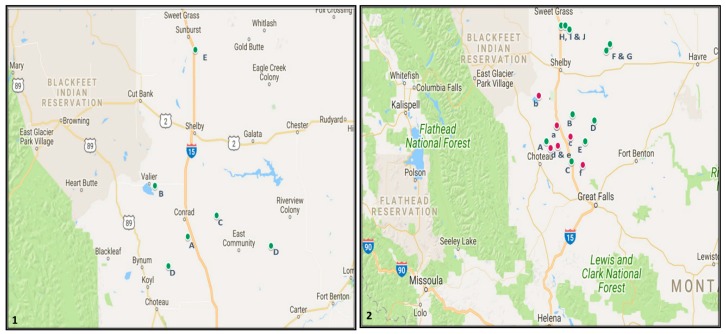
Montana State showing field sites: (**1**) trap type and placement and (**2**) lure type. Green and pink circles indicate field pea and lentil fields, respectively.

This study took place in 2016 in five counties (Pondera, Toole, Teton, Liberty and Chouteau) in the Golden Triangle of Montana, USA (Figure 1). Field variation between counties was due to either availability of fields or to driving time to reach fields. In some instances, the same field was used for different experiments (Table 1) and a minimum of 50 m distance was maintained between treatment fields to avoid interference. In the Golden Triangle region field peas are usually seeded in April to May at 170–225 kg seeds/ha. Average field pea yields in Montana varied from 2245 to 3400 kg/ha [28]. All the counties in this study were known to have had high levels of *S. lineatus* infestation in recent years.

### 2.2. Pheromone Lures

Two types of pheromone lures—a gray rubber septum and membrane, each impregnated with 4-methyl-3,5-heptanedione, were obtained from ChemTica Internacional SA (Costa Rica). Each type of lure contained 10 mg of pheromone, emitting the active ingredient at 0.1 mg/day. Septa were prepared by depositing 100 µL of a 0.1 mg/µL hexane solution into the cup of each septum and the hexane was allowed to evaporate at room temperature over 4 h. The membrane release devices were prepared by heat sealing the required amount of pheromone in a circular plastic pouch (1 cm internal diameter) using a custom sealing device. The pheromone slowly evaporated through the proprietary plastic. All lures were shipped in aluminum pouches and stored at 4 °C until use.

### 2.3. Experiment #1: Trap Type

**Figure 2:**
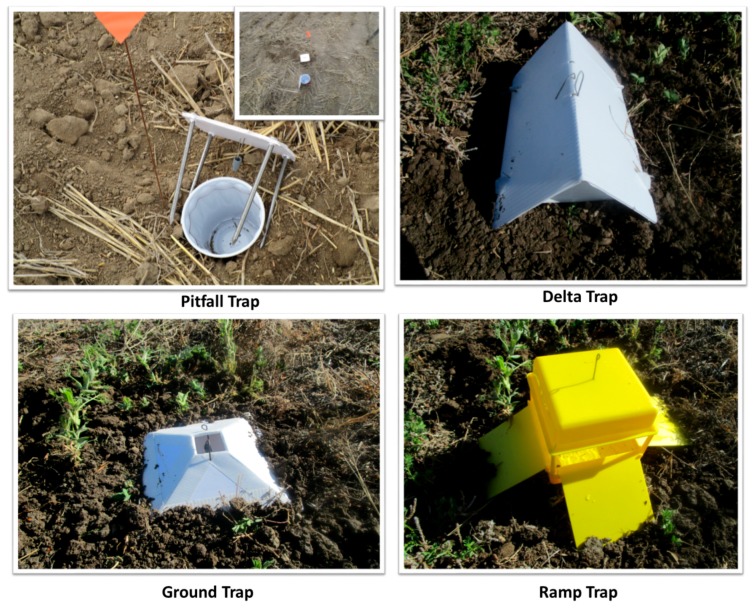
Trap types (pitfall, delta, ground and ramp traps) used to evaluate the most effective trap design in capturing the pea leaf weevil, *Sitona lineatus*.

Experiment #1 (Trap Type) was conducted at six sites, clustered in three areas (southern, central and northern) in the Golden Triangle region, with three fields in the central area, two fields in the southern area and one field in the northern area (Table 1). The clustering was based on similarities of climate (e.g., temperature, moisture and humidity). Four different trap types were examined: (1) pitfall trap; (2) ground trap; (3) delta trap; and (4) ramp trap (Figure 2). Delta traps were obtained from Great Lakes IPM Inc. (Vestaburg, MI, USA) and ramp traps from ChemTica Internacional SA (Costa Rica). Ground traps were constructed in our laboratory based on a design from previous studies [25,26]. A red solo cup (diameter 6.5 cm; height 12.5 cm) served as a pitfall trap.

Traps were arranged in a randomized complete block design with three replicates per treatment. Traps without pheromone lures were used as controls. There was a control for each trap type. They were installed in all experimental fields in the first week of April 2016 and were placed at least 25 m from the field edge, 25 m apart and 25 m between blocks. Trap positions were rotated biweekly to minimize positional effects on trap catch. The traps were monitored weekly until the end of July, and the number of weevils caught per trap was recorded on each sample date. No foliar pesticide applications were made in the experimental areas.

### 2.4. Experiment #2: Lure Type

Experiment #2 (Lure Type) compared rubber septa versus membranes as pheromone dispensers and the pitfall trap type was used for experiment. This experiment was run in both field peas and lentils to see if the lures were also effective in lentils, a minor host crop of the pest [29]. The experiment was conducted at 16 field sites (10 pea and 6 lentil fields), in Pondera, Teton, Chouteau and Toole Counties (Table 1). Field sites were clustered based on three geographical areas (southern, central and northern) in the Golden Triangle region, with one to five fields in each cluster (Table 1). Each field represents a replicate in each cluster and treatments were arranged in a randomized complete block design with two subreplicates per treatment. Traps without a pheromone lure were used as controls. The distances between traps within and between subreplicates were as described above. Trap positions were rotated and data collected as previously described for Experiment #1.

### 2.5. Experiment #3: Trap Placement

Experiment #3 (Trap Placement) was conducted at three fields in the central area of the Golden Triangle (Table 1). Each field was considered a replicate since all fields were from the same geographical area. Three traps were placed in each field, with one trap at the southern end and one trap at the northern end of the field. A third trap without pheromone lure was considered as control, and was rotated from northern to southern side of the field to avoid possible side and placement effects on trap captures. Traps were installed during the first week of April and the experiment continued through June 2016. Lures were changed every six weeks.

### 2.6. Statistical Analysis

The data were found to be non-normally distributed and therefore, nonparametric one-way analysis of variance (Kruskal–Wallis test) was used to determine the effects of trap type, lure type, and trap placement on *S. lineatus* total trap capture over the entire trapping period. A Mann–Whitney *U*-test was used as a post-hoc test for multiple comparisons between the means followed by a Bonferroni correction to adjust the probability (α = 0.01). The data were analyzed using the software statistical package R 2.15.1 [30].

## 3. Results

### 3.1. Experiment #1 Trap Type

**Figure 3:**
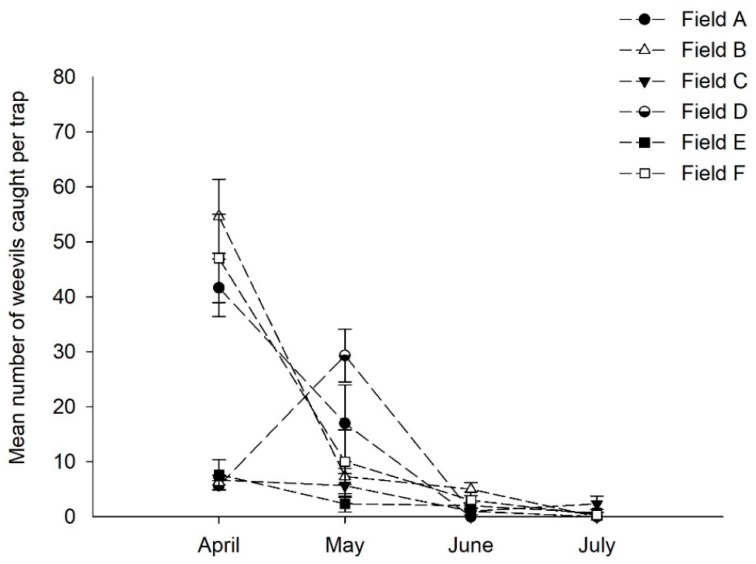
*Sitona lineatus* adults captured by pitfall traps baited with pheromone at six experimental sites in the Golden Triangle, Montana.

Regardless of experimental site, more *S. lineatus* adults were caught in traps baited with pheromone lures than in control traps (Figure 3). There were no significant differences in adult captures in control traps among fields (southern: *χ^2^* = 4.56; *df* = 3; *p* = 0.21, Kruskal–Wallis test; central: *χ^2^* = 3.11; *df* = 3; *p* = 0.38, Kruskal–Wallis test). Most adults were caught early in spring, with nearly 90% of captures occurring in April or May, 2016. The average number of adults caught per trap and collection period declined over the growing season (Figure 3). Among experimental sites, adults were caught in higher numbers in northern followed by central and southern areas of the Golden Triangle region.

**Figure 4:**
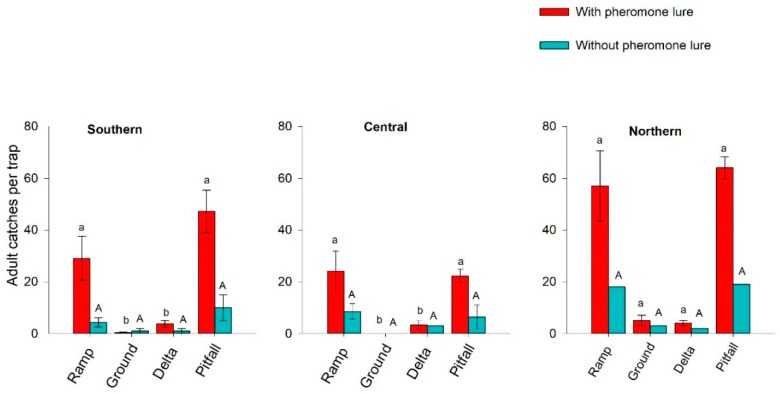
Mean total captures per trap ± S.E. of *Sitona lineatus* adults in different traps in pea fields at southern, central and northern clusters of the Golden Triangle, Montana, from the first week of April to the end of July 2016. Mean values within bars bearing the same upper-case or lower-case letters in each cluster are not significantly different (Mann–Whitney *U*-tests followed by Bonferroni correction [α = 0.01]).

Trap type had a significant impact on adult captures at all study clusters (southern: *χ^2^* = 18.27; *df* = 3; *p* < 0.001, Kruskal–Wallis test; central: *χ^2^* = 26.67; *df* = 3; *p* < 0.0001, Kruskal–Wallis test; northern: *χ^2^* = 8.56; *df* = 3; *p* = 0.03, Kruskal–Wallis test). At the southern and central field clusters, a significantly higher number of adults were caught in pitfall and ramp traps baited with pheromone lures than in ground or delta traps, while there were no significant differences in adult captures between pitfall and ramp traps or between ground and delta traps (Figure 4). Similarly, at the northern field clusters, pitfall and ramp traps caught higher adult populations than ground and delta traps but without significant differences (Figure 4). Across fields, the range of mean number of *S. lineatus* captures per trap and collection period for pitfall, ramp, ground and delta traps varied from 11–64, 9–57, 0.27–5 and 1–4, respectively, irrespective of study clusters. Although ramp and pitfall traps were equally effective, the pitfall trap was selected for all further experiments because it is less expensive and easily fabricated.

### 3.2. Experiment #2: Lure Type

**Figure 5:**
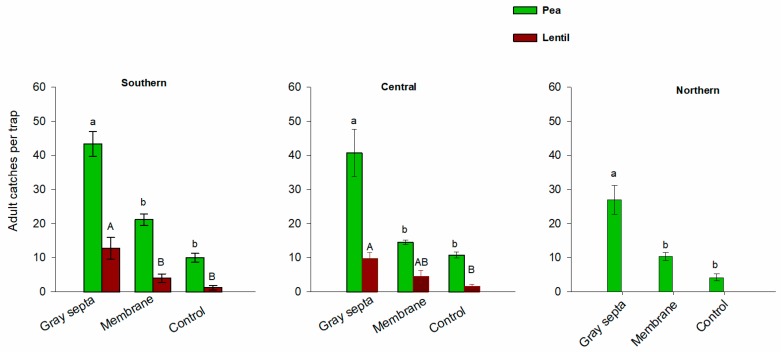
Mean total captures per pitfall trap ± S.E. of *Sitona lineatus* adults in pea and lentil fields at southern, central and northern clusters of the Golden Triangle, Montana, from the first week of April to the end of July, 2016. Mean values within bars bearing the same upper-case or lower-case letters in each cluster are not significantly different (Mann–Whitney *U*-tests followed by Bonferroni correction [α = 0.01]).

Lure type significantly affected mean *S. lineatus* adult trap captures in the pea fields, with rubber septa lures capturing more weevils than traps with membrane lures or unbaited controls at all experiment clusters (southern: *χ^2^* = 10.38; *df* = 2; *p* < 0.01, Kruskal–Wallis test; central: *χ^2^* = 13.12; *df* = 2; *p* < 0.01, Kruskal–Wallis test; northern: *χ^2^* = 10.07; *df* = 2; *p* < 0.01, Kruskal–Wallis test) (Figure 5). Average seasonal total catches in the southern clusters were 43.37 ± 3.66 per pitfall trap baited with rubber septa, 21.12 ± 1.77 baited with membrane lure and 10.00 ± 1.26 for unbaited traps. In the central clusters, mean adult captures were 40.75 ± 6.89 per pitfall trap baited with rubber septa, 14.50 ± 0.60 baited with membrane lure and 10.75 ± 0.85 for unbaited traps. The corresponding values at the northern clusters were 27.00 ± 4.25, 10.33 ± 1.17 and 4.16 ± 1.01, respectively (Figure 5). Similarly, in the lentil fields, significantly higher numbers of adults were recorded for the pitfall traps baited with rubber septa compared to those baited with membrane lures and unbaited traps at all experiment clusters (southern: *χ^2^* = 7.95; *df* = 2; *p* = 0.02, Kruskal–Wallis test; central: *χ^2^* = 9.99; *df* = 2; *p* = 0.01, Kruskal–Wallis test) (Figure 5).

### 3.3. Experiment #3: Trap Placement

We found no significant differences in *S*. *lineatus* adults captured in pitfall traps placed in northern and southern parts of pea fields (*χ^2^* = 1.19; *df* = 2; *p* = 0.27, Kruskal–Wallis test). However, there was a tendency to capture relatively higher numbers in the southern parts (48.33 ± 24.89 [SE]) compared with the northern parts (23.68 ± 7.89 [SE]).

## 4. Discussion

The development of a semiochemical-based trapping system would be useful for monitoring *S. lineatus* populations and would allow growers to develop Integrated Pest Management (IPM) strategies for controlling this pest [18,19]. Monitoring of *S. lineatus* activity at overwintering sites will help growers to prolong or adjust field pea planting dates in areas of high weevil density [31]. In addition, capture in pheromone-baited traps within pea fields in the spring could be used to better target foliar applications of insecticides against weevils before oviposition [31,32].

Trap type is known to impact adult captures of many insects [26]. Pitfall, ground, ramp, delta, funnel, and sticky traps are all commercially available and have been tested against a variety of insect pests [25,26,27]. For example, Reddy et al. [25,26] demonstrated that pheromone-baited ground traps were more effective in catching adults of old-house borer *Hylotrupes bajulus* (L.) (Coleoptera: Cerambycidae) and banana root borer *Cosmopolites sordidus* (Germar) (Coleoptera: Curculionidae) than ramp and pitfall traps because the adults prefer to walk when they reach the host or the pheromone source. In contrast, Valles et al. [33] found that bucket traps consistently outperformed sticky, box, and *Heliothis* traps in capturing and retaining pickleworm adults (*Diaphania nitidalis* [Stoll] [Lepidoptera: Crambidae]) because the adults typically fly. For *S. lineatus*, St. Onge et al. [21] showed that pitfall traps baited with pheromone caught significantly more *S. lineatus* adults than solo cup, yellow cone, yellow bucket or green unitrap designs. Nielsen and Jensen [20] found that cone traps were more effective than yellow sticky traps for pea leaf weevil in northern European alfalfa or clover fields. To the best of our knowledge, other trap types such as ground, ramp, and delta have not been evaluated previously against *S. lineatus* adults. Optimization of trap features for adult population is hence timely, and the optimized methods can be used in other parts of the world where this widespread pest is a problem.

Our data support the results of St. Onge et al. [21], indicating that pitfall traps are likely the best trap design for monitoring *S. lineatus* adults. We found that ramp traps were as effective as pitfall traps but they are comparatively more expensive and difficult to use than pitfall traps. Moreover, in Montana pea fields, strong winds often occur, which make ramp traps less suitable as they are difficult to secure to the ground. Delta and ground traps were found to be less suitable to catch adults in all our study sites. The low efficiency of these traps could be due to the fact that overwintered adults often prefer to walk or crawl rather than flying while feeding on susceptible crop plants during spring [11,34], thereby hindering adult capture in delta and ground traps installed above ground. However, adults are known to be good fliers, especially during their two flight activity periods (a post-teneral flight in late summer and a post-diapausal flight during the spring), which are triggered by photoperiod [11,34].

Pitfall traps baited with gray rubber septa were much more attractive to *S. lineatus* adults than those baited with the membrane lures or unbaited traps. Rubber septa may have provided better protection from oxidation or polymerization of the ketone than the membrane lures [35]. However, there are occasions on which membrane lures have been found to be superior to other lure types [36]. For instance, Malo et al. [36] found the membrane lure to be better than septa lure to attract the fall armyworm, *Spodoptera frugiperda* (Smith) (Lepidoptera: Noctuidae).

Trapping location within a crop field can be an important factor affecting trap capture. Insect response to trap placement has been described in various species, such as in pickleworm *D. nitidalis* [33], jasmine moth *Palpita unionalis* (Hübner) (Lepidoptera: Pyralidae) [37], and banana root borer *C. sordidus* [25] and *Tuta absoluta* (Meyrick) (Lepidoptera: Gelechiidae) [38]. In our study, traps placed at the southern part of the pea field caught nearly twice as many insects than did traps placed at the northern part of the field, although the difference was not significant. The lack of significant impact of trap placement in our study was likely due to few replications which resulted in a lot of variation in mean trap captures. Nevertheless, this result is consistent with the findings of Quinn et al. [32], who recorded higher populations of *S. lineatus* adults in pheromone traps at the southern compared to the northern parts of a field, as could be expected because adults prefer sites in a field with higher temperatures and sunlight.

## 5. Conclusions

In summary, this study shows that trap design, lure type, and trapping location are all important features that affect the response of *S. lineatus* adults to pheromone-baited traps. Pitfall traps baited with gray rubber septa placed at the southern end of pea fields were more effective than the other traps, locations, and lure dispensers examined. These findings should be taken into consideration while developing a pea leaf weevil monitoring system. In addition, wind tunnel/olfactometer studies are required to confirm the efficacy of host/plant-based volatile compounds against *S. lineatus* [17,39]. In this context, faba bean has been reported to be a highly susceptible crop [4,19] that can be used as border trap crop for adults. It would be interesting to understand the chemical compounds from this plant so that they can possibly be used along with the pheromone compounds to have a synergistic effect in attracting *S. lineatus*, and could help in mass trapping or even mating disruption of *S. lineatus*. Additional mark and recapture studies may be also needed to determine the maximum radius of attraction to determine the number of traps and lures required for a given cropping area. It will be also worthwhile to determine the pheromone threshold levels for *S. lineatus*. Development and optimization of a trapping system for *S. lineatus* would be a useful tool in providing information for monitoring and precise assessments of adult emergence and timing of the control measures.

## Figures and Tables

**Table 1:** Description of field sites for pheromone trapping of *Sitona lineatus* at the Golden Triangle, Montana, 2016.

Experiments	Field Site	Trap Deployed Date	Geographical Coordinates		Total Cultivated Area (Hectares)	Golden Triangle Area
			Latitude	Longitude		
Trap type	Field A	4 April 2016	N 48°06.325′	W 111°56.060′	53	Central
	Field B	6 April 2016	N 48°18.463′	W112°12.086′	55	Central
	Field C	4 April 2016	N 48°11.398′	W 111°40.952′	259	Central
	Field D	11 April 2016	N48°06.351′	W111°19.477′	18	Southern
	Field E	18 April 2016	N 47°59.500′	W 112°05.436′	132	Southern
	Field F	16 May 2016	N 48°50.347′	W 111°51.713′	61	Northern
Lure type	*Pea*					
	Field A	4 April 2016	N 48°06.325′	W111°56.060′	53	Central
	Field B	4 April 2016	N 48°11.398′	W 111°40.952′	259	Central
	Field C	18 April 2016	N 47°59.500′	W 112°05.436′	132	Southern
	Field D	11 April 2016	N 48°06.351′	W111°19.477′	18	Southern
	Field E	11 April 2016	N 47°59.220′	W 111°28.076′	259	Southern
	Field F	19 April 2016	N 47°50.518′	W 111°41.286′	16	Southern
	Field G	14 April 2016	N 48°39.628′	W 111°08.545′	216	Northern
	Field H	22 April 2016	N 48°50.347′	W 111°51.713′	61	Northern
	Field I	16 April 2016	N 48°50.475′	W 111°47.769′	97	Northern
	Field J	22 April 2016	N 48°18.458′	W 111°55.529′	129	Northern
	*Lentil*					
	Field A	11 April 2016	N 48°06.831′	W 111°55.475′	32	Central
	Field B	2 May 2016	N 48°46.131′	W 110°54.192′	55	Central
	Field C	4 April 2016	N 48°01.785′	W 111°42.553′	20	Southern
	Field D	9 April 2016	N 47°57.360′	W 111°54.560′	129	Southern
	Field E	13 April 2016	N 47°56.0541′	W 112°01.069′	65	Southern
	Field F	11 May 2016	N 47°48.452′	W 111°30.321′	111	Southern
Trap placement	Field A	4 April 2016	N 48°06.325′	W 111°56.060′	53	Central
	Field B	6 April 2016	N 48°18.463′	W 112°12.086′	55	Central
	Field C	10 April 2016	N 48°11.398′	W 111°40.952′	259	Central
